# Diagnostics of inflammaging in relation to sarcopenia

**DOI:** 10.3389/fpubh.2023.1162385

**Published:** 2023-07-03

**Authors:** Barbara Morawin, Anna Tylutka, Filip Bielewicz, Agnieszka Zembron-Lacny

**Affiliations:** ^1^Department of Applied and Clinical Physiology, Collegium Medicum University of Zielona Gora, Zielona Góra, Poland; ^2^Student Research Group, University of Zielona Gora, Collegium Medicum University of Zielona Gora, Zielona Gora, Poland

**Keywords:** aging, cytokines, inflammation, sarcopenia, lifestyle exercise, skeletal muscle

## Abstract

One of the theories about aging focuses on the immune response and relates to the activation of subclinical and chronic inflammation. This study was designed to investigate the relationship between inflammation and sarcopenia and to evaluate the influence of lifestyle on the inflammatory profile. Finally, therapeutic strategies to counteract the pathophysiological effect of skeletal muscle aging were also indicated. One hundred seventy-three individuals aged 71.5 ± 6.8 years were divided into two groups: sarcopenia and probable sarcopenia (*n* = 39) and no sarcopenia (*n* = 134). Sarcopenia was assessed according to the algorithm of the European Working Group on Sarcopenia in the older adults 2. C-reactive protein (CRP) (*p* = 0.011) and CRP/albumin ratio (*p* = 0.030) as well as IL-1β (*p* = 0.002), cfDNA (*p* < 0.001) and bilirubin levels (*p* = 0.002) were significantly higher in the sarcopenia group as opposed to the no sarcopenia group. No significant differences were observed between groups in the concentration of TNFα (*p* = 0.429) and IL-6 (*p* = 0.300). An inverse correlation was found between gait speed and cfDNA (*r*_s_ = −0.234, *p* < 0.01) and IL-1β (*r*_s_ = −0.263, *p* < 0.01). The ROC analysis of cfDNA, CRP, IL-1β and bilirubin ranged from 0.6 to 0.7, which confirms the association between sarcopenia and inflammatory mediators and indicates high clinical usefulness of cfDNA and bilirubin in sarcopenia prediction. We also indicated a link between inflammation and fitness level in the older adult thereby providing evidence that lifestyle exercise should be a key therapeutic strategy in sarcopenia prevention.

## 1. Introduction

One of the theories on aging focuses on the immune response and relates to the activation of subclinical and chronic inflammation (1, 2). Inflammaging is expressed by the release of a large number of inflammatory mediators which are produced to repair tissue-level damage such as pro-inflammatory cytokines IL-1, IL-2, IL-6, IL-8, IL-12, IL-13, IL-15, IL-18, IL-22, IL-23, tumor necrosis factor α (TNFα), interferon-γ (IFN-γ), anti-inflammatory cytokines IL-1Ra, IL-4, IL-10, transforming growth factor (TGF-β1), and also lipoxin A4 and heat shock proteins (2–4). Most human studies have concentrated on the pro-inflammatory cytokines IL-6 and TNFα contribute to the damaging catabolic effects of inflammation on aging muscle fibers, which leads to skeletal muscle degradation, sarcopenia acceleration, and eventually, a decline in physical performance (5–8). These mechanisms are not still fully understood, but studies have remarked that an elevated level of pro-inflammatory cytokines disturbance anabolic and catabolic processes (9). Inflammation is related with an increased risk of age-related diseases, such as cardiovascular diseases, type 2 diabetes, sarcopenia, osteoporosis, cognitive decline, and frailty syndrome, all of which share a common inflammatory pathogenesis (10–12).

Extensive scientific evidence has demonstrated the relation of low physical performance with subclinical and chronic inflammation which can lead to physical disabilities and, in extreme cases, to hospitalization and even death of the older adult. Physical inactivity in various age groups has been a serious challenge for health systems of late. Older adult people pose a particularly complex problem because those with sarcopenia have been shown to be more likely to falls and death when compared to the older adult without sarcopenia (13). Adverse outcomes of sarcopenia cause to seek new therapies to prevent the degenerative loss of skeletal muscles mass, quality and strength, which significantly affect the quality of life of the geriatric population (14, 15). It is considered that physical activity is one of the most effective intervention which alleviates inflammation and delays sarcopenia (16). It is noteworthy that the physical activity type and frequency as well as individual response to exercise have a significant impact on the inflammatory response (17). Resistance exercises increase muscle mass and strength while endurance exercises improve aerobic capacity and metabolic regulation (14, 18). However, the combination of endurance and resistance training has been demonstrated to produce the best results by lowering the levels of inflammatory mediators, mainly C-reactive protein (CRP), IL-6 and TNFα, which are also strong predictors of all-cause mortality risk in 80-year-old population (19). Moreover, exercise training has been reported to enhance anti-inflammatory capacity (20, 21), to improve neutrophils chemotaxis (22), to augment natural killer (NK) cells cytotoxicity and to increase T lymphocytes proliferation (23), as well as improving post-vaccination response (24). Daily physical activity which particularly affects immunity and dramatically declines with age has not been widely investigated yet. The majority of studies concentrated on the effects of exercise training intervention or pharmacological and stem cell therapies (25–27). Recently we demonstrated that a 10-month physical intervention significantly reduced the symptoms of sarcopenia through the changes to the body composition and physical performance and *via* the improvement in TNFα-related apoptosis (18). Furthermore, we revealed that an increased intake of anti-inflammatory diet ingredients and daily physical activity attenuate inflammaging and counteract the adverse effects of aging (2). The present study focused firstly, on the evaluation of the major features of inflammaging in relation to sarcopenia, secondly, on the impact of lifestyle exercise on inflammatory profile and, thus, indications of therapeutic strategies to counteract the pathophysiological effect of skeletal muscle aging.

## 2. Materials and methods

### 2.1. Participants

The study was conducted with the participation of 212 older adults people from the University of the Third Age. The medical documentation provided by the primary care physician and health history questionnaire were the basis for assessing the current health status and lifestyle of the respondents (28). Similar medical care and age > 60 years and were inclusion criteria for the study. The presence of the following diseases in subjects: acute infectious diseases, uncontrolled hypertension and/or diabetes, oncologic diseases, neurodegenerative diseases, musculoskeletal disturbances and an implanted pacemaker were exclusion criteria. Thirty-nine subjects were excluded from further participation due to high blood pressure above 180/110 mmHg, uncontrolled diabetes, serious knee injury and hip and malaise. Eventually, 173 subjects aged 71.5 ± 6.8 years (females *n* = 136, males *n* = 37) were included in the study. The subjects were categorized into two groups based on the WHO protocol: the youngest-old aged 60–74 years (*n* = 123) and the middle-old/oldest-old aged 75–90 years (*n* = 50) (29, 30). Additionally, in order to compare inflammatory processes, the subjects were divided into two groups: the first group included the older adult people diagnosed with probable sarcopenia, confirmed sarcopenia, severe sarcopenia (*n* = 39), and the second group of the subjects without sarcopenia (*n* = 134). The European Working Group on Sarcopenia in Older People 2 (EWGSOP2) algorithm was used to assess sarcopenia among the older adult people (31). Some of the subjects took antihypertensive drugs (58%) and hypolipidemic drugs (24%) as well as anticoagulants, including anti-platelet agents (8%). Each participant signed an informed consent to participate in the project. The Bioethics Commission at Regional Medical Chamber Zielona Gora, Poland (No. 01/66/2017) approved the protocol of the research, in accordance with the Helsinki Declaration.

### 2.2. Body composition and muscle mass

The bioelectrical impedance method was used to estimate body mass (BM), body composition fat-free mass (FFM) and fat mass (FM) by means of Tanita Body Composition Analyzer MC-980 (Japan). The analyzer was calibrated according to the manufacturer’s guidelines before each test session. The measurements were taken between 7:00 and 9:00 am, prior to blood sampling.

### 2.3. Analysis of sarcopenia

The clinical algorithm was used for sarcopenia diagnosis according to the European Working Group on Sarcopenia in Older People (EWGSOP2). The algorithm takes into account several stages of the procedure: sarcopenia case-finding, assessment, confirmation, and severity determination (31). The initial assessment of sarcopenia was made on the basis of the reduction in skeletal muscle contraction strength, the reduction of muscle mass was used to confirm sarcopenia diagnosis and physical performance, including the 6-min walk test (6MWT), was assessed as an indicator of severity. A hand dynamometer, KERN type MAP130 (Germany) was used to measure the hand grip strength. Hand grip strength was measured twice in a sitting position with arms in their sides. The subjects had to squeeze the dynamometer as hard as possible, and the results were given in kilograms. To determine, the cut-off points for sarcopenia, the EWGSOP2 recommendations were used, according to which low muscle strength was <16 kg for women and < 27 kg for men. To confirm sarcopenia among the subjects, the following indices were used: appendicular skeletal muscle mass (ASM <15 kg for women and < 20 kg for men) and appendicular skeletal muscle mass index (ASMI <5.5 kg/m^2^ for women, ASMI<7.0 kg/ m^2^ for men) (31–33). Gait speed was measured during 6MWT, which was performed following the standards of European Respiratory Society and American Thoracic Society (34). The subjects had to walk as far as possible in 6 min, but subjects were allowed to self-pace and to rest as needed. The total distance covered was recorded and gait speed was calculated by the following equation: gait speed (m/s) = total distance(m)/360 s. Following to EWGSOP2 recommendations, the cut-off point for sarcopenia was 0.8 m/s (31). Additionally, activity classification based on gait speed according to Middelton et al. (35) was used to characterize the activity of the older adult. Following to this division, the gait speed within the range of 1.0 to 1.3 m/s classified the older adult as active, the gait speed <1.0 m/s classified them as inactive and the gait speed >1.3 m/s classified them extremely fit.

### 2.4. Blood sampling

Peripheral blood was collected from the median cubital vein in participants who were fasting and physically inactive for at least 48 h prior to blood sampling. Blood samples collected in S-Monovette-EDTA K2 anticoagulant tubes (Sarstedt, Germany) were subjected for hematology analysis, while blood collected in S-Monovette clotting activator tubes (Sarstedt, Germany) was applied for biochemical analysis. Prior to centrifugation, blood collected in a clotting activator tube was allowed to clot for 45 min. After 45 min, the blood collected was centrifuged for 10 min at 3000 g and + 8°C. Serum aliquots were stored in a low-temperature freezer that maintains a temperature at −80°C until biochemical analysis and inflammatory markers were performed.

### 2.5. Hematological variables

The 3 diff BM HEM3 Biomaxima hematology analyzer (Poland) was used to perform hematology analysis including the white blood cell system (white blood cells WBC, lymphocytes LYM, granulocytes GRA), the red blood cell system (red blood cells RBC, hemoglobin HB, hematocrit HCT) and platelets PLT.

### 2.6. Biochemical variables

The triglycerides (TG), total cholesterol (TC), low-density lipoprotein (LDL) and high-density lipoprotein (HDL) were determined using BM200 Biomaxima (Poland). The non-HDL cholesterol was calculated by subtracting HDL from the total cholesterol concentration. Oxidized low-density lipoprotein (oxLDL) was determined by using ELISA kits from SunRed (China) with detection limit 30.3 ng/mL. Diaglobal spectrophotometer (Germany) was used for measurement of the glucose level. Total bilirubin was determined by using BM200 Biomaxima (Poland).

### 2.7. Inflammatory variables

C-reactive protein (CRP) level was measured using a high-sensitivity assay in duplicate by means of commercial kit from DRG International (United States) with the detection limit of 0.001 mg/L. Albumin was determined by using BM200 Biomaxima (Poland). C-reactive protein to albumin ratio (CRP/albumin) was calculated as CRP (mg/L) divided by albumin level (g/L) (36). ELISA kits from SunRed Biotechnology Company (China) were used to measure levels of tumor necrosis factor α (TNFα), interleukin 6 (IL-6), interleukin 1β (IL-1β) and the kits had detection limits of 2.782 pg./mL, 1.867 pg./mL and 28.384 pg./mL, respectively. Quant-iTTM high-sensitivity DNA assay kit was used to determine the concentration of the total circulating fragments of DNA (cell free DNA) and readings were made on a Qubit fluorometer (Invitrogen, Carlsbad, CA, United States). The analysis was carried out in duplicate, and the mean of the two measurements was recorded as the final value. The intra-assay CV for the Quant-iTTM DNA high-sensitivity assay was <2%.

### 2.8. Statistical analysis

Statistical analyzes were performed using Statistica 13.1 (StatSoft Inc., Tulsa, OK, United States) and R 4.0.3 software (37). The Shapiro–Wilk test was used to evaluate the normality of the distributions. The significant differences in mean values between the groups (sarcopenia and no-sarcopenia) were assessed by the one-way ANOVA. If the normality was violated, the Kruskal-Wallis non-parametric test was used. Additionally, eta-squared (*η*^2^) based on function in RStudio was used to measure of effect size which is indicated as having no effect if 0 ≤ *η*^2^ < 0.01, a small effect if 0.01 ≤ *η*^2^ < 0.06, a moderate effect if 0.06 ≤ *η*^2^ < 0.14, and a large effect if *η*^2^ ≥ 0.14. Spearman’s rank correlation coefficient (*r*_s_) or Pearson correlation coefficient (r) were used to investigate the relationships between biochemical and inflammatory variables. The optimal clinical stratification thresholds (cut-off values) were based on Receiver Operating Characteristic (ROC) curves by calculating the Youden index. The results are expressed as mean value ± standard deviation (SD) or median (Me). Statistical significance was set at *p* < 0.05.

## 3. Results

### 3.1. Body composition and muscle mass

Body mass index (BMI) values ranged from 18.8 to 38.8 kg/m^2^ in 60–74 years group, and from 18.6 to 35.0 kg/m^2^ in the subjects over 75 years of age (Table 1). Among all the respondents, only 32% of the subjects had a normal weight (BMI 18.5–24.930 kg/m^2^), while the majority of the subjects were either overweight (BMI 25.0–29.930 kg/m^2^) or obese (BMI ≥30 kg/m^2^), where the value was 49 and 19%, respectively. The BMI value was strongly correlated with fat mass (FM) (r = 0.806, *p* < 0.0001), FM% (r = 0.523, *p* < 0.0001) and fat-free mass (FFM) (r = 0.463, *p* < 0.0001). We recorded the predicted significant differences between men and women in such indicators as BM, FM%, MM and FFM, which confirms anthropometric gender differences. Men were characterized by a lower content of FM% and higher MM and FFM. There were no significant differences in FM between the sexes, although there was a trend toward higher values detected in women than in men. Table 1 likewise presents the breakdown of anthropometric and body composition data in the group of men and women in terms of the age: 60–74 and 75–90 years. Our study found a significantly higher body weight in men aged 60–74 compared to men over 75, mainly due to the higher content of lean body mass. Contrary to expectations, this study did not find significant differences in these markers in women, only a trend toward higher values was observed in the 60–74 age group. However, we did not show significant differences in the content of adipose tissue in either group. These results may suggest that with age, men are more likely to experience changes in body composition and, above all, loss of muscle mass than women.

**Table 1:** Anthropometrics and body composition.

	Age 60–74 yr			Age > 75 yr			Females 60–74 yr. vs. Females >75 yr. *p* value	Males 60–74 yr. vs. Males >75 yr. *p* value
	Females *n* = 106 Mean ± SD (Me)	Males *n* = 17 Mean ± SD (Me)	Females vs. Males *p* value	Females *n* = 30 Mean ± SD (Me)	Males *n* = 20 Mean ± SD (Me)	Females vs. males *p* value		
Age [yr]	67.4 ± 3.5 (68.0)	70.1 ± 2.0 (70.0)	0.004	79.0 ± 3.6 (78.0)	82.5 ± 4.9 (81.0)	0.008	<0.001	<0.001
Body mass [kg]	67.4 ± 9.9 (66.9)	83.1 ± 9.8 (80.2)	<0.0001	65.8 ± 9.4 (64.9)	73.8 ± 12.9 (72.6)	0.028	0.445	0.024
Height [cm]	160.7 ± 5.0 (160.7)	170.2 ± 5.9 (169.0)	<0.001	155.3 ± 5.8 (155.9)	165.5 ± 8.6 (167.0)	<0.001	<0.001	0.076
BMI [kg/m^2^]	26.2 ± 3.3 (25.9)	28.7 ± 2.9 (28.7)	0.003	27.3 ± 4.2 (27.1)	27.0 ± 4.0 (27.3)	0.854	0.128	0.187
FM [kg]	23.9 ± 6.5 (22.8)	21.6 ± 5.8 (21.4)	0.202	23.2 ± 6.3 (23.8)	18.9 ± 7.4 (19.5)	0.044	0.945	0.249
FM%	34.7 ± 5.4 (35.0)	25.7 ± 4.4 (26.8)	<0.0001	34.9 ± 5.4 (35.9)	24.4 ± 7.3 (24.9)	<0.0001	0.541	0.233
MM [kg]	41.6 ± 4.1 (41.2)	58.3 ± 4.7 (57.3)	<0.0001	40.1 ± 3.8 (40.6)	52.8 ± 7.6 (53.4)	<0.001	0.108	0.016
FFM [kg]	43.8 ± 4.3 (43.4)	61.4 ± 4.9 (60.3)	<0.0001	42.3 ± 4.0 (42.8)	55.5 ± 8.0 (56.2)	<0.001	0.109	0.016

BMI body mass index, FM fat mass, MM muscle mass, FFM fat free mass, SD standard deviation, Me median.

### 3.2. Assessment of sarcopenia

77.5% of the subjects were characterized by normal muscle strength, muscle mass and physical fitness. Probable sarcopenia was demonstrated in 18.5% of the older adults (*n* = 32), confirmed sarcopenia in 2.3% (*n* = 4), and severe sarcopenia in 1.7% (*n* = 3). Severe sarcopenia was observed only in people over 75 years of age. The first criterion in sarcopenia assessment is to determine the level of muscle strength. Over 3/4 of all our subjects achieved the expected grip strength values. Higher values of muscle strength were demonstrated in no sarcopenia group than in sarcopenia group (Table 2). Positive correlations were found between the strength of the dominant hand and the muscle mass of the upper limbs (*r* = 0.585, *p* < 0.0001), ASM (*r* = 0.579, *p* = 0.0001), the gait speed (*r* = 0.390, *p* = 0.0001) and albumin (*r*_s_ = 0.271, *p* < 0.01). In our study group, 91.9% of women and 91.9% of men reached the correct ASM value according to Cruz-Jentoft et al. (31). Only 2% of the individuals did not obtain the correct ASMI values <5.5 kg/m^2^ and < 7.0 kg/m^2^ in women and men, respectively. Interestingly, no significant differences were noticed in the ASM and ASMI indices between the two groups. Sarcopenia group and no sarcopenia one covered the distance of 325.00 ± 83.77 m and 454.96 ± 65.37 m, respectively. There were significant differences between sarcopenic and non-sarcopenic groups in 6MWT and the gait speed results. A negative correlation between age and gait speed (*r* = −0.564, *p* < 0.0001) was observed, i.e., the gait speed decreased with age. About 94.2% of the respondents walked with a gait speed above 0.8 m/s, which according to EWSGOP2 is an important point in the algorithm for assessing the severity of sarcopenia (31). As Table 3 shows, there is a significant difference in grip strength (*p* < 0.0001) and gait speed (*p* < 0.0001) between the group of women with and without sarcopenia. Similar relationships were observed in men, moreover, men showed significant differences in ASMI (*p* = 0.041). Significant differences between muscle strength, ASM and ASMI were noted between men and women in the sarcopenia group and those without sarcopenia. Interestingly, the data in this table show no differences in gait speed between the sexes.

**Table 2:** Assessment of sarcopenia.

	Sarcopenia *n* = 39 Mean ± SD (Me)	No sarcopenia *n* = 134 Mean ± SD (Me)	Sarcopenia vs. no sarcopenia *p* value
Grip strength [kg]	14.74 ± 4.60 (14.55)	24.83 ± 6.59 (23.30)	<0.0001
ASM [kg]	18.11 ± 2.86 (17.70)	18.79 ± 3.61 (17.80)	0.439
ASMI [kg]	7.16 ± 0.83 (7.18)	7.17 ± 1.04 (6.85)	0.287
6MWT [m]	325.00 ± 83.77 (337.50)	454.96 ± 65.37 (455.00)	<0.0001
Gait speed [m/s]	0.90 ± 0.23 (0.94)	1.26 ± 0.18 (1.26)	<0.0001

ASM appendicular skeletal muscle mass, ASMI appendicular skeletal muscle mass index, 6MWT the 6-min walk test, SD standard deviation, Me median.

**Table 3:** Assessment of sarcopenia by sex.

	Sarcopenia *n* = 36			No sarcopenia *n* = 118			Females sarcopenia vs. females no sarcopenia *p* value	Males sarcopenia vs. males no sarcopenia *p* value
	Females *n* = 28 Mean ± SD (Me)	Males *n* = 11 Mean ± SD (Me)	Females vs. males *p* value	Females *n* = 108 Mean ± SD (Me)	Males *n* = 26 Mean ± SD (Me)	Females vs. males *p* value		
Grip strength [kg]	13.4 ± 2.1 (13.5)	18.5 ± 7.3 (18.7)	0.008	22.2 ± 3.6 (22.0)	35.6 ± 5.2 (36.7)	<0.0001	<0.0001	<0.00001
ASM [kg]	16.8 ± 1.5 (17.0)	21.4 ± 2.8 (21.7)	<0.001	17.4 ± 2.0 (17.3)	24.5 ± 3.4 (24.1)	<0.0001	0.213	0.017
ASMI [kg/m^2^]	6.9 ± 0.7 (6.9)	7.9 ± 0.9 (7.9)	0.003	6.8 ± 0.7 (6.7)	8.6 ± 0.9 (8.4)	<0.0001	0.151	0.041
6MWT [m]	336.4 ± 91.4 (350.0)	303.2 ± 65.2 (305.0)	0.320	459.3 ± 66.8 (457.5)	436.9 ± 56.6 (445.0)	0.214	<0.0001	<0.0001
Gait speed [m/s]	0.93 ± 0.25 (0.097)	0.84 ± 0.17 (0.85)	0.320	1.28 ± 0.19 (1.27)	1.22 ± 0.16 (1.24)	0.214	<0.0001	<0.0001

ASM appendicular skeletal muscle mass, ASMI appendicular skeletal muscle mass index, 6MWT the 6-min walk test, SD standard deviation, Me median.

### 3.3. Hematological variables

In all subjects, white blood cell (WBC) count and red blood cells (RBC) count were recorded to fall within the referential range, i.e., 4.0–10.2 10^3^/μLand 4.0–5.5 10^6^/μL, respectively. No statistically significant differences in WBC, granulocytes and platelets counts were observed between sarcopenic and no sarcopenic groups (Table 4). In the white blood cell system, only the lymphocytes count was significantly lower in the group diagnosed with sarcopenia. While in the red blood cell system, all indicators (RBC, HB, HCT) were significantly lower in the sarcopenia group. The value of η^2^ indicated a large effect of sarcopenia on red blood cells, hemoglobin and hematocrit.

**Table 4:** Hematological variables.

	Sarcopenia *n* = 39 Mean ± SD (Me)	No sarcopenia *n* = 134 Mean ± SD (Me)	Sarcopenia vs. No sarcopenia *p* value	*η*^2^
WBC [10^3^/μL]	6.1 ± 1.9 (5.4)	6.4 ± 1.7 (6.2)	0.141	0.006
Lymphocytes [10^3^/μL]	1.7 ± 0.5 (1.7)	2.2 ± 0.8 (2.1)	<0.0001	0.085
Granulocytes [10^3^/μL]	3.9 ± 1.7 (3.4)	3.8 ± 1.4 (3.6)	0.147	0.000
RBC [10^6^/μL]	4.3 ± 0.5 (4.4)	4.8 ± 0.4 (4.8)	<0.0001	0.199
HB [g/dL]	12.9 ± 1.3 (13.0)	13.8 ± 0.9 (13.8)	<0.0001	0.114
HCT [%]	35.9 ± 3.6 (36.0)	39.2 ± 2.7 (39.3)	<0.0001	0.187
Platelets [10^3^/μL]	251.0 ± 68.2 (240.0)	249.9 ± 62.8 (247.0)	0.895	0.000

WBC white blood cells, RBC red blood cells, HB hemoglobin, HCT hematocrit, *η*^2^ is a measure of effect size, SD standard deviation, Me median.

### 3.4. Biochemical variables

The concentrations of triglyceride (TG) and total cholesterol (TC) were found to be at similar levels in all subjects. The mean values for TG were 136.7 ± 35.3, while for TC 230.1 ± 46.4 mg/dL. Approximately 28% of all the subjects exceeded the reference values for TG >150 mg/dL and 76% of them exceeded the reference values for TC >200 mg/dL. There was no significant difference in the concentration of non-HDL between the groups. Significant differences were observed in the levels of LDL and HDL, where the concentration of LDL was significantly higher in the sarcopenia group, while the concentration of HDL was significantly higher in the no sarcopenia group (Table 5). The value of η^2^ indicated a moderate effect of sarcopenia on the concentration of LDL and HDL. Some correlations were noted between TG and FM% (*r*_s_ = 0.402, *p* < 0.0001) i.e., the larger the adipose tissue, the higher the concentration of TG. An elevated glucose level is known as a biomarker of aging and nutritional status, and it is associated with alterations in metabolic and hormonal function, including altered expression of cellular insulin receptors and glucose transporter units in target tissues (2). There was a significant difference in glucose concentration between both groups but the value of *η^2^* indicated small effect of sarcopenia on the glucose concentration. The glucose level in the sarcopenia group ranged from 63.5 to 125.0 mg/dL, while in the no sarcopenia group the values amounted to 75.5–125.0 mg/dL. Some participants (9%) demonstrated glucose values above 115 mg/dL but none of the participants were diagnosed with diabetes. The concentration of total bilirubin as a biomarker of aging (38) was within the reference values (<1 mg/dL) in all older adult people, but was significantly higher in the sarcopenia group compared to the non-sarcopenic group. The *η^2^* value showed a moderate effect of sarcopenia on bilirubin level.

**Table 5:** Biochemical variables.

	Sarcopenia *n* = 39 Mean ± SD (Me)	No sarcopenia *n* = 134 Mean ± SD (Me)	Sarcopenia vs. No sarcopenia *p* value	*η*^2^
TG [mg/dL]	135.3 ± 42.2 (142.9)	137.1 ± 33.2 (130.3)	0.400	0.001
TC [mg/dL]	222.4 ± 55.2 (216.3)	232.4 ± 43.5 (232.4)	0.240	0.000
LDL [mg/dL]	107.7 ± 35.9 (111.0)	81.0 ± 27.6 (79.2)	<0.0001	0.111
HDL [mg/dL]	65.9 ± 16.2 (68.0)	79.0 ± 19.5 (81.7)	<0.0001	0.109
non-HDL [mg/dL]	156.5 ± 48.1 (156.1)	155.1 ± 48.3 (147.8)	0.878	0.001
oxLDL [ng/mL]	331.6 ± 389.00 (170.7)	628.1 ± 525.7 (489.0)	0.008	0.034
Glucose [mg/dL]	93.5 ± 15.3 (92.2)	99.3 ± 11.7 (99.7)	0.012	0.035
Bilirubin [mg/dL]	0.24 ± 0.17 (0.24)	0.16 ± 0.33 (0.07)	0.002	0.050

TG triglycerides, TC total cholesterol, LDL low-density lipoprotein, HDL high-density lipoprotein, non-HDL cholesterol calculated by subtracting the HDL value from a TC, oxLDL oxidized low density lipoprotein, *η*^2^ is a measure of effect size, SD standard deviation, Me median.

### 3.5. Inflammatory variables

**Figure 1:**
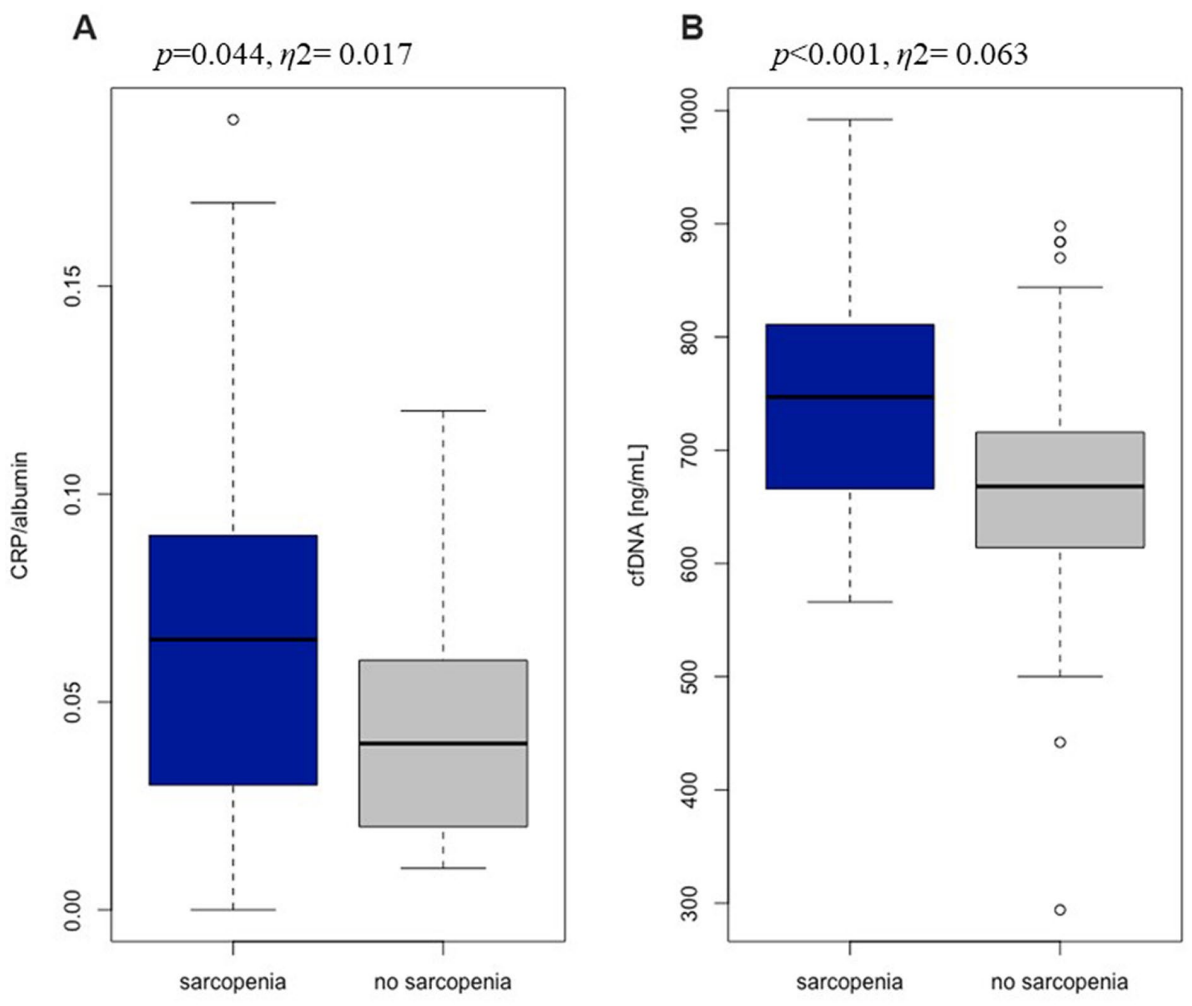
The ratio of C-reactive protein to albumin **(A)** and the level of cell free DNA **(B)** in sarcopenia group (*n* = 39) and no sarcopenia group (*n* = 134).

The level of CRP ranged from 0.07 to 8.4 mg/dL and fell within the reference range (<3 mg/dL) in 74,5% of the subjects. A significantly higher level of CRP was recorded in the group with sarcopenia compared to without sarcopenia, which clearly indicates the relationship of systemic inflammation and skeletal muscle degradation (Table 6). In all subjects, albumin was within references value (37.0–53.0 g/L for women, 42.0–55.0 g/L for men). However, sarcopenia group was characterized by significantly lower albumin levels than the group of undiagnosed sarcopenia. Albumin level was shown to decrease with increasing age by approx. 0.1 g/L per year. Inflammation, especially high levels of the pro-inflammatory cytokines IL-6 and TNFα, is a major factor in lowering albumin levels (39). CRP is a highly sensitive positive marker of inflammation, while albumin is a negative marker of inflammation. Due to these relationships, the CRP/albumin ratio may be a more sensitive indicator for determining inflammation than the CRP and albumin analyzed separately (40). The CRP/albumin ratio was significantly higher in our study sarcopenic group (Figure 1A). Some of the classic biomarkers of aging include cytokines such as TNF α, IL-1β, and IL-6. There were no significant differences between our two study groups in TNFα and IL-6 levels. The concentration of IL-1β was significantly higher in the group with sarcopenia than without the condition and negatively correlated with the gait speed (*r*_s_ = −0.263, *p* < 0.01) which, in turn, points to the relation of inflammation with the functional impartment in old age. Moreover, a positive correlation was demonstrated between IL-1β and TNFα (*r*_s_ = 0.372, *p* < 0.0001) and IL-1β and cfDNA (*r*_s_ = 0.241, *p* < 0.01). The mean concentration of cfDNA (749.5 ± 102.7 ng/mL) in the group with sarcopenia was significantly higher compared to the non-sarcopenic one (679.6 ± 109.4 ng/mL, Figure 1B). A negative correlation was found between cfDNA and the gait speed (*r*_s_ = −0.234, p < 0.01). The value of η^2^ demonstrated a large effect of sarcopenia on the changes in cfDNA. This is consistent with the study by Jylhävä et al. (41) who reported that cfDNA could serve as a novel biomarker of inflammaging.

**Table 6:** Inflammatory variables.

	Sarcopenia *n* = 39 Mean ± SD (Me)	No sarcopenia *n* = 134 Mean ± SD (Me)	Sarcopenia vs. No sarcopenia *p* value	*η*^2^
CRP [mg/L]	3.23 ± 2.45 (3.00)	2.14 ± 1.73 (1.58)	0.011	0.032
Albumin [g/L]	43.761 ± 2.70 (43.78)	46.53 ± 2.61 (46.28)	<0.0001	0.114
TNFα [pg/mL]	112.83 ± 81.10 (91.64)	94.62 ± 60.38 (91.82)	0.429	0.002
IL-6 [pg/mL]	116.96 ± 94.08 (81.18)	106.13 ± 89.19 (68.80)	0.298	0.000
IL-1β [pg/mL]	1406.74 ± 1120.23 (864.08)	755.11 ± 353.90 (712.91)	0.002	0.049

CRP C-reactive protein, TNFα tumor necrosis factor α, IL-6 interleukin 6, IL-1β interleukin 1β, *η*^2^ is a measure of effect size, SD standard deviation, Me median.

### 3.6. Assessment of the specific variables in the diagnosis of sarcopenia

The results of the ROC analysis of the indicators which are likely to be important in the diagnosis of the inflammaging in sarcopenia, i.e., cfDNA, CRP and IL-1β ranged from 0.6 to 0.7, which can be considered as a potential diagnostic value for clinical prognosis for older patients. The optimal threshold values corresponded to 736 ng/mL for cfDNA, 0.08 for CRP/albumin ratio, 0.20 mg/dL for bilirubin and 1260.7 pg./mL for IL-1β (Table 7). Surprisingly, the highest AUC, indicating both high sensitivity and specificity, was observed for bilirubin. The highest specificity was observed for IL-1β (90.3%), CRP/albumin ratio (86.7%) and bilirubin (79.7%), which indicates a low level of false positive results during diagnostic procedure. However, sensitivity values for IL-1β and CRP/albumin ratio were fairly low (40.7 and 42.8, respectively). This means that more than half of the patients with sarcopenia may show lower levels of the markers than the estimated threshold. Consequently, a combined, simultaneous analysis of several factors seems to be the best solution.

**Table 7:** The statistical characteristics of the ROC curve for the univariate logistic model for specific variables.

Variables	AUC	Cut-off value	Sensitivity (%)	Specificity (%)
cfDNA [ng/mL]	0.689	736	58.3	75.4
CRP [mg/dl]	0.643	2.7	55.8	73.4
Albumin [g/L]	0.237	45.02	25.0	28.5
CRP/Albumin	0.588	0.08	42.8	86.7
TNFα [ng/mL]	0.545	35.1	96.9	15.3
IL-6 [pg/mL]	0.562	33.2	96.6	20.8
IL-1β [pg/mL]	0.690	1260.7	40.7	90.1
Bilirubin [mg/dL]	0.719	0.207	72.7	79.2

cfDNA cell-free DNA, CRP C-reactive protein, TNFα tumor necrosis factor α, IL-6 interleukin 6, IL-1β interleukin 1β, AUC the area under the curve, cut-off value the optimal threshold value for clinical stratification.

## 4. Discussion

Inflammaging appears to be of key importance to our understanding of the aging process, and anti-inflammion seems to be one of the processes which underlie longevity (3). Thus, inflammaging should be analyzed more thoroughly in order to intervene in a timely and multidimensional and appropriate preventive and therapeutic approaches (2, 42). A healthy lifestyle practiced throughout life has been reported to have an influence on longevity and the aging process. The new field of lifestyle medicine which focuses primarily on nutrition and physical activity can significantly affect the quality of life of the older adult (43).

One of the theories of aging is based on the subclinical chronic inflammation the markers of which is CRP induced by proinflammatory cytokines, especially IL-6 (44). An increase in CRP expression was observed in inflammatory conditions such as rheumatoid arthritis, cardiovascular diseases, cancer and sepsis (45). While CRP increases in chronic inflammation, albumin decreases, so, the CRP/albumin ratio may be a more sensitive marker to predict inflammation than either CRP or albumin levels analyzed separately (40). CRP/albumin ratio is used as a prognostic mediator for several cancers such as hepatocellular lung cancer, newly diagnosed pancreatic cancer, renal cancer or ovarian cancer (44). So far, the prognostic significance of CRP/albumin ratio in the aging and inflammatory processes in sarcopenia has not been reported. Our previous research showed that that nutritional frailty and slow gait speed (<0.8 m/s) were closely associated to high CRP/albumin ratio (2). Our present study is the first to have demonstrated a significantly higher CRP/albumin ratio in sarcopenia compared to no sarcopenia older adult, and the ratio could be used as one of the novel biomarkers of inflammation in sarcopenia. It was also the first time that ROC curves had been analyzed in sarcopenic patients and the cut-off point for the CRP/albumin ratio was estimated at 0.08.

Pro-inflammatory cytokines, especially TNFα, IL-6 and IL-1β, are amid the most widely investigated inflammatory indicators in studies on the process of aging (46). Despite extensive research, the role of low-grade chronic inflammation in age-related sarcopenia is still unclear (47). In our study, no significant differences were identified in TNFα levels between the sarcopenic and non-sarcopenic groups. In the study by Hammami et al. (48), pro-inflammatory biomarkers of frailty like TNF-α or IL-8 were assessed and the analysis of ROC curves performed on a group of 141 people aged>65 years showed that the TNF-α level of 22.71 was the predictive threshold of frailty, with an AUC of 0.66 (*p* = 0.016; 95% CI [0.54–0.79]). Our study appears to be the first to have assessed the TNFα cut-off value (35.1 ng/mL) in sarcopenic patients and the value is consistent with the results reported by Hammani et al. (48). Many studies have already shown a relationship between the decline in skeletal muscle strength and IL-6 level (5, 49–52). Contrary to expectations, the relationship was not confirmed by the outcomes obtained in this study. Surprisingly, we did not detect any significant differences in IL-6 concentration between sarcopenic and non-sarcopenic groups either, although a significant role of IL-6 in sarcopenia has already been reported by a large number of studies. Even though we did not record such differences, we observed the trend toward higher IL-6 values in the sarcopenia group. In addition, the AUC (0.562) for IL-6 indicated its lowered diagnostic utility in inflammation in our sarcopenic group. The lack of differences between the groups may result from the large statistical dispersion of the test results. It should also be emphasized that IL-6 is a multi-directional cytokine, produced rapidly and transiently in response to infections and tissue damage and it is continuously expressed in a variety of cell populations in a range of diseases (53). Notwithstanding the application of strict exclusion criteria for some diseases, other comorbidities and factors affecting IL-6 concentration cannot be excluded. Among other things, the use of pro- and anti-inflammatory drugs can modulate inflammatory processes (54). In a meta-analysis by Bano et al. (13) sarcopenia was reported to be connected with higher CRP values, but not with higher concentrations of IL-6 and TNFα. On the one hand, IL-6 plays a physiopathological part in skeletal muscles but, on the other hand, muscle-derived IL-6 stimulates anabolic pathways regulating muscle growth (55). It is important to distinguish between the acute and chronic effects of proinflammatory cytokines. Chronic inflammation can affect catabolic processes in skeletal muscles and lead to a decline in physical performance (56). Scheede-Bergdahl et al. (57) demonstrated that IL-6 was not the best pro-inflammatory clinical biomarker in cancer cachexia. In their study, IL-1β was shown to be better associated with the clinical features of a cachectic state such as weakness, anorexia, weight loss and sarcopenia than IL-6 (57). IL-1α and IL-1β are believed to belong to the mediators of inflammation-related skeletal muscle atrophy. IL-1α and IL-1β can act directly on skeletal muscles, triggering catabolic programs that lead to the degradation of myofibrillar proteins. IL-1β may interfere with myogenesis in myoblasts by blocking the action of insulin-like growth factor (IGF) (58). In this study, significantly higher values of IL-1β were detected in the sarcopenia group. Our review of the available literature proved that the relationship of sarcopenia and IL-1β had been scarcely investigated. Our research may indicate a potential use of IL-1β as an indicator of sarcopenia (AUC for IL-1β was 0.690), however, more research should be conducted to confirm this relationship. In this study IL-1β concentration was found to be negatively correlated with the gait speed (*r*_s_ = −0.263, *p* < 0.01). These data suggest that inflammation may adversely affect the functional ability of older people. The experimental studies conducted so far suggest that the severity of inflammation is an important mechanism related to sarcopenia (5–8). However, despite numerous studies, the role of cytokines in age-related sarcopenia is yet to be clarified (14).

Many biomarkers of aging have already been identified but new potential ones are constantly being sought. Cell-free DNA appears to be the most promising, largely because of its ease of sampling, but there little data on how cfDNA changes with age (59). Several reports have proved that cfDNA increases with age (59, 60). Theo et al. (59) also showed that cfDNA could be used as a health condition predictor. Cellular damage triggers cfDNA release into the circulation, and the plasma cfDNA level reflects the extent of the tissue damage and the resulting inflammation (60). The available literature provides very little data on the relationship between sarcopenia and cfDNA. Jylhava et al. (60) and Swarup et al. (61) demonstrated that higher levels of cfDNA were related with systemic inflammation and increased fragility and other degenerative diseases of muscles and neurons. This is consistent with our study findings as we observed a significantly higher value of cfDNA in the older adult with sarcopenia compared to the non-sarcopenic group. Likewise, the value of the η2 index indicated a considerable influence of sarcopenia on cfDNA level. In addition, we recorded correlations between cfDNA and IL-1β (*r*_s_ = 0.241, *p* < 0.01), and the gait speed (*r*_s_ = −0.234, *p* < 0.01). This underlines cfDNA relationship with inflammation and, indirectly, also with the functional performance of the older adult. To date, most research has focused on the determination of the cut off and AUC values for circulating cell free mtDNA (ccf-MtDNA)in older adult sarcopenia and the AUC value of 0.726 was reported (62). By contrast, our research focused on cfDNA and its potential application in the early diagnosis of sarcopenia might be indicated by a high value of AUC (0.689). According to these data, cfDNA can be used as a new, easily measurable marker of inflammation in sarcopenic older adult people.

Our study found an association between sarcopenia and certain inflammatory mediators as CRP, albumin, CRP/albumin ratio, IL-1β, and cfDNA. For the first time, we demonstrated a significantly higher CRP/albumin ratio and an elevated level of cfDNA in the group with diagnosed sarcopenia compared to the older adult without sarcopenia, thereby indicating that the two parameters could be used as novel biomarkers of sarcopenia. Nevertheless, more research is needed to clarify the applicability of CRP/albumin ratio and cfDNA in the diagnosis of inflammation development in sarcopenic older adult people. In addition, the results of the ROC analysis of the indicators which may be of some importance in the diagnosis of inflammation in sarcopenia, i.e., cfDNA, CRP and bilirubin ranged from 0.6 to 0.7, which can be considered as a potential diagnostic value for clinical prognosis for older adult and could be helpful in early diagnosis of sarcopenia. This is an important point for future investigation because despite the promising results, many questions still remain unanswered. The available literature is lacking in reports which included the ROC analysis performed for the aforementioned indicators. Therefore, it is very important to continue the research for our findings to be confirmed. Apart from the assessment of the inflammation associated with sarcopenia, the purpose of our study was also to evaluate the influence of lifestyle exercises on the inflammatory profile, and thus to identify therapeutic strategies to counteract the pathophysiological effects of skeletal muscle aging. The sarcopenia group was characterized by lower muscle strength and gait speed as well as increased levels of inflammatory markers compared to the group without sarcopenia. Higher levels of IL-1β, and cfDNA were associated with a lower gait speed in the older adult. Older adult without diagnosed sarcopenia were more physically fit than people from the sarcopenia group, because as many as about 42.5% of people from this group achieved a speed above 1.3 m/s, while un the sarcopenia group no person achieved such a gait speed. According to Studenski (63), the older adult, who walk at speeds exceeding 1.3 m/s, are characterized by a high physical fitness level, whereas, according to the classification by Middelton et al. (35) the gait speed above 1.3 m/s classifies the older people as extremely fit. The gait speed is considered as a clinical marker of functional capacity in the older adult (64). Busch et al. (64) suggested that a lower gait speed was related to age and education, but it was especially associated with modifiable factors such as instrumental activities of daily living (IADL) impairment, lack of physical activity and cardiovascular diseases. Additionally, studies by Laudiso et al. (65) showed a relationship between the quality of life of the older adult and muscle strength. It should be noted that the reduced quality of life of the older adult, in addition to reduced physical fitness, may be significantly affected by the increased content of adipose tissue, which is often accompanied by a loss of muscle mass (66). Older adult people diagnosed with sarcopenia or sarcopenic obesity are particularly vulnerable to a decrease in quality of life (67). Therefore, the priority should be to maintain functional efficiency, the ability to independently perform basic life activities affecting the quality of life (64). Lifestyle exercises can contribute to the reduction of some symptoms of sarcopenia (18). Visser et al. (68) showed that people leading a healthy lifestyle had a 10.6% slower decrease in gait speed observed within the 3-year study period. The researchers believe that a healthy lifestyle positively affected physical, mental, cognitive and social functioning until old age (68). Peterson et al. (69) found that those who regularly exercised at the start of the study were less likely to develop weakness over a 5-year period compared with those who led a sedentary lifestyle. Exercise was reported to reduce the level of some frailty markers such as TNFα, IL-6, CRP (70). In our study, we noted lower values of inflammatory markers (CRP, CRP/albumin ratio, TNFα, IL-6, IL-1β, cfDNA) in the no-sarcopenia group, as opposed to the sarcopenia group. Lifestyle modifications through the introduction of regular physical activity are key to healthy aging and should be the target of therapeutic strategies to reduce mobility decline and the risk of sarcopenia (71).

As is the case with most studies, our research is also subject to several limitations. The study included a relatively small number of participants diagnosed with sarcopenia or potential sarcopenia. Another limitation of the study is the unequal proportion of sexes, as well as the lack of information on participants’ lifetime exposure to pathogens that may have disproportionately affected the inflammatory markers analyzed. Despite the exclusion of people with certain diseases, we cannot fully exclude the influence of other diseases to which older people are exposed. Hence, further randomized trials involving large cohorts are required to provide stronger evidence for predicting patient outcomes. In spite of these limitations, the study certainly adds to our understanding of the relationship between sarcopenia and inflammation, as well as the role of physical activity as one of the important therapeutic strategies used to prevent skeletal muscle aging.

## 5. Conclusion

In order to effectively prevent and predict sarcopenia, it is essential to identify the appropriate index and the corresponding optimal cut-off level. The results of this study indicate a association between sarcopenia and inflammatory mediators and the high clinical usefulness of cfDNA and bilirubin in the prediction of sarcopenia. We also demonstrated a link between inflammation and physical fitness in the older adult, and that lifestyle exercise should be a key therapeutic strategy in sarcopenia prevention. A better understanding of the inflammatory mechanisms involved in skeletal muscle aging could increase our understanding of sarcopenia prevention.

## Data availability statement

The raw data supporting the conclusions of this article will be made available by the authors, without undue reservation.

## Ethics statement

The studies involving human participants were reviewed and approved by Bioethics Commission at Regional Medical Chamber Zielona Gora, Poland (No. 01/66/2017). The patients/participants provided their written informed consent to participate in this study.

## Author contributions

BM and AZ-L: conceptualization, methodology, writing–original draft preparation, visualization, and funding acquisition. BM, AT, and AZ-L: validation and resources. BM, AT, FB, and AZ-L: formal analysis, writing–review and editing, and data curation. BM and AT: investigation. AZ-L: supervision. BM: project administration. All authors contributed to the article and approved the submitted version.

## Funding

This work was supported by funds from the University of Zielona Gora and from the National Science Center Poland (No 2016/21/N/NZ7/03329).

## Conflict of interest

The authors declare that the research was conducted in the absence of any commercial or financial relationships that could be construed as a potential conflict of interest.

## Publisher’s note

All claims expressed in this article are solely those of the authors and do not necessarily represent those of their affiliated organizations, or those of the publisher, the editors and the reviewers. Any product that may be evaluated in this article, or claim that may be made by its manufacturer, is not guaranteed or endorsed by the publisher.
